# Lifestyle factors associated with childhood obesity: a cross-sectional study in Shanghai, China

**DOI:** 10.1186/s13104-014-0958-y

**Published:** 2015-01-17

**Authors:** Liangli Li, Tingting Shen, Li Ming Wen, Min Wu, Ping He, Youfa Wang, Weidong Qu, Hui Tan, Gengsheng He

**Affiliations:** Department of Nutrition and Food Hygiene, School of Public Health, Key Laboratory of Public Health Safety, Ministry of Education, Fudan University, Shanghai, People’s Republic of China; Department of Maternal, Child and Adolescence Health, School of Public Health, Key laboratory of Public Health Safety, Ministry of Education, Fudan University, Shanghai, People’s Republic of China; School of Public Health, University of Sydney, Sydney, Australia; Health Promotion Service, Sydney Local Health District, Sydney, NSW Australia; Department of Social and Preventive Medicine, School of Public Health and Health Professions, Joint Appointments, School of Medicine and Biomedical Sciences, University at Buffalo, State University of New York (SUNY), New York, USA; Department of Environmental Health, School of Public Health, Key Laboratory of Public Health Safety, Ministry of Education, Fudan University, Shanghai, People’s Republic of China

**Keywords:** Obesity, Screen time, Physical activity, Sugar-sweetened beverage

## Abstract

**Background:**

Limited research has been conducted to investigate factors associated with overweight and obesity of children in China, where obesity has been increasing. This study investigated associations of lifestyle factors with overweight or obesity among Chinese primary school-aged children.

**Methods:**

A cross-sectional survey was conducted with 2400 children aged 6–12 from 11 primary schools. Children completed a self-administered questionnaire assisted by their parents at home. The survey included questions on self-reported height and weight, screen time, physical activity, modes of travel to/from school, and dietary habits. Multilevel models were conducted to examine factors associated with overweight or obesity.

**Results:**

15.6% of children were overweight and 11.2% were obese; nearly 80% of children spent ≤2 hrs./day either on physical activities or screen time. Compared with those spent >3 hrs./day on screen time, children who spent ≤2 hrs./day or between 2-3 hrs./day were significantly less likely to be obese after adjusting for other variables (AOR = 0.34, 95% CI: 0.20-0.60, P < 0.01; or AOR = 0.41, 95% CI: 0.20-0.84, P = 0.02 respectively). Children spent ≤2 hrs./day on screen time were less likely to become overweight or obesity, compared with >3 hrs./day (AOR = 0.62, 95% CI: 0.38-0.99, P < 0.05).

**Conclusions:**

Screen time is independently associated with childhood obesity, and needs be focused for obesity prevention in school-aged children in China.

## Background

Obesity has become a global public health issue and is widely recognized as a key risk factor for coronary heart disease, hypertension, diabetes and many other health problems [1]. The prevalence of childhood and adolescent obesity in the World Health Organization (WHO) European Region has risen over the past few decades [1]. In 2010, 43 million children were reported to be overweight and obese, and 92 million children were at risk of overweight [2]. The prevalence of childhood overweight and obesity increased worldwide from 4.2% in 1990 to 6.7% in 2010 [3]. Similarly, for the past 20 years in China, the rate of overweight and obesity of school-aged children has increased by 4–6 times in highly developed cities [4]. In 2010, it was estimated that 9.9% of Chinese school-aged children and adolescents were overweight and an additional 5.1% were obese, representing an estimated 30.43 million individuals [5].

Factors related to overweight and obesity of children have been well documented [6–8], including dietary behaviors, physical activity, screen time, modes of journey to school and sleep duration. A previous research found that increases in Sugar-sweetened beverages (SSB) consumption led to decreases in milk and fruit juice intake from early childhood into adolescence [8]. Besides, children’s time spent on physical activity and screen time are now recognized as important factors related to children’s weight status and other health outcomes [9]. However, few studies of this kind have been conducted among Chinese children, which could hamper the development of effective obesity prevention programs in China. Based on Song et al’s study, unhealthy dietary behaviors, sedentary life style were significantly associated with overweight and obesity of school-aged children in China [10]. Another study by Zhang et al. found that watching TV/videos and doing homework before and after school were significantly and positively associated with Body Mass Index (BMI) in boys, but not in girls [11]. Whether screen time, physical activity, SSB and snack consumptions are associated with overweight and obesity of Chinese school-aged children remains unclear.

This study aimed to explore the associations of lifestyle factors such as screen time, physical activity, and food consumption with childhood obesity in Shanghai, China.

## Methods

### Study design

A school-based, cross-sectional survey was conducted with 11 elementary schools located in Hongkou District, Shanghai, China between June and September 2012. The study was approved by the Ethical Review Board of Fudan University School of Public Health.

Multi-stage cluster sampling was used to select the study sample. The sample size of 3000 children was estimated based on an approximate rate of 25% of overweight/obesity in children reported in other school studies in Shanghai, China [12], taking into account an estimated response rate of 70%. Eleven primary schools were randomly selected from 56 schools located in Hongkou District. For each grade, we selected about 50 children (25 boys) from the school. A total of 3515 students aged between 6 and 12 years old were included in the study.

Students who were not willing to participate in the survey or did not complete all survey questions were excluded from this study. A total of 2400 students provided complete data and were included in the analyses, which represented a response rate of 68%. There were no statistically differences in any of demographic characteristics including maternal education, household income and maternal occupation between those included in and excluded from the study.

### Data collection

The self-administered questionnaire including 33 questions were handed out to each student in class. Students were asked to complete the questionnaire together with their parents at home. Informed consents were obtained from all participants’ parents.

### Survey measures

The majority of the survey questions were adapted from the 2010 New South Wales Schools Physical Activity and Nutrition Survey (SPANS) [13] in which most of key measures have been tested for their reliability and validity. The questionnaire collected information on socio-demographic indicators, physical activity (including modes of transport to/from school, sports participation), screen time (television watching and computer use), and dietary behaviors (including SSB and snack consumptions).

Self-reported weight and height were collected. We used the international standard age adjusted BMI (BMI = weight/height^2^) to classify children’s weight status as ‘non-overweight’ (including underweight), ‘overweight’ and ‘obese’ [14]. ‘Non-overweight’ combined with ‘overweight’ was also regrouped as ‘non-obese’. To examine the validity of self-reported weight status we also conducted a validation study with a sub-group of 65 students. We had compared the weight status derived from self-reported or measured weight and height according to the WHO Child Growth Standards [14] (correlation coincident for weight status = 0.74, P < 0.01).

Parents along with their child were asked to estimate the time spent by the child in a range of activities including both physical activities and screen time in a usual school week, separately for weekdays (outside school hours) and weekends. Physical activities include organized games, sports as well as unorganized physical activities. Information on screen time was collected by asking how long their child spent on watching TV, videos or using computer. The responses were classified as ‘≤2 hrs./day’, ‘2-3 hrs./day’ or ‘>3 hrs./day’ for physical activity or screen time respectively. Children’s modes of travel to/from school were recorded as ‘active transport’ (walking/cycling) and ‘inactive transport’ (public transport, car or other methods).

Information about students’ dietary habits and patterns was collected using a set of short questions. Children along with their parents were asked to report the child’s daily consumption of fruits and vegetables, the frequency of consuming fast food, SSB (including soft and sweetened drinks and sports drink) or snacks using food frequency questions (FFQ). Snacks were listed in the questionnaire as ‘sweets, meat seafood and eggs, grain, beans, vegetable and fruits, dairy and dairy products, nuts, potatoes, ice-cream’. Information on snacks was categorized into three groups: “Healthy Snacks” (including vegetables and fruits, dairy, nuts), “Energy dense, nutrient poor” (including sweets, ice-cream, chips), “Others” (including biscuits, bread, sausages, dried bean curd). The frequencies of snacks were recorded as ‘Hardly eat’, ‘1-2 times/wk.’, ‘3-4 times/wk.’ and ‘>5 times/wk.’. SSB frequencies were classified into ‘≤2 cps/wk., 2-5 cps/wk., 5-7 cps/wk. and >7 cps/wk.’.

Socio**-**demographic information was also collected including child age, gender, school, class, maternal education level, employment status and household income. Maternal education was classified into ‘Primary school, High school and College/University or above’. Household income was divided as ‘≤3000, 3001–8000, and ≥8001 in Chinese Yuan ($1 = 6.1 Yuan)’.

### Statistical analyses

All data were analyzed using SAS software version 9.2 with a P < 0.05 indicating statistical significance. Descriptive analyses were conducted in reporting distributions of study variables of interest. The study outcomes were weight status (non-overweight, overweight and obese, or obese). Chi-square tests were used in bivariate analyses. Relationships between study factors and the outcomes were examined with Spearman Correlations (Spearman Nonparametric Correlations).

Considering the cluster sampling and unequal probability of selection, school and individual levels were further included in multilevel models. Variables that were found to be associated with overweight & obesity or obesity on bivariate analyses (P < 0.25) were entered into the multilevel models. ‘School’ was included in the model as a random effect. Adjusted odds ratios (AORs) with 95% confidence intervals (CI) were calculated as a measure of association. Model A was adjusted for age, gender, household income, modes of travel to/from school; Model B was adjusted for those included in Model A + physical activity; Model C was adjusted for those included in Model A + snacks/screen time; Model D was adjusted for those included in Model A + SSB; and Model E was adjusted for those included in Model A + snacks/screen time + SSB + physical activity. Collinearity between the study variables was also tested while conducting multilevel models.

## Results

### Characteristics of study participants

Table 1 shows the characteristics of the participants in the study. Overall, 15.2% of children were overweight and 10.9% were obese. Boys were more likely to be overweight or obese than girls 21.8% vs. 9.4%; and 14.4% vs. 8.0% respectively). About half children (49.2%) went to school by car or bus, while 50.8% children walk or cycle to school daily. On a usual weekday children spent an average of 0.81 ± 0.90 hour on screen time outside school. Time spent on physical activities was about 1.10 ± 1.13 hours. On a usual weekend-day children spent more time on screen time 2.40 ± 1.77 hours or physical activities 2.33 ± 1.67 hours than on a weekday. There was significant gender difference in daily screen time (boy: 1.31 ± 1.06, girl: 1.22 ± 0.90, P = 0.03). The mean consumption of SSB was (3.19 ± 4.46) cps/wk. (1 cup = 250 ml). Significantly, boys consumed more SSB than girls (boy: 3.48 ± 4.82 cps/wk. girl: 2.89 ± 4.04 cps/wk., P = 0.01). Besides, there were significant gender differences in consumption of “Energy dense, nutrient poor” and “Other” snacks (P = 0.03, and P = 0.01 respectively).

**Table 1:** **Characteristics of participating children aged 6–12 years in Shanghai, China, 2012**

	**Total N = 2400**	**Girls N = 1190**	**Boys N = 1210**	**P** * **(chi-square test)**
	**n(%)**	**%**	**%**	
**Weight status** ^**△**^				**<0.01**
Non-overweight	1706(73.2)	82.6	63.8	
Overweight	364(15.6)	9.4	21.8	
Obese	262(11.2)	8.0	14.4	
**Maternal education Level**				0.17
Primary school	203(8.8)	7.7	9.8	
High school	1094(47.4)	47.3	47.5	
College/University or above	1012(43.8)	45.0	42.7	
**Household income** **(RMB/month)**				0.12
≤3000	412(20.6)	19.0	22.2	
3001-8000	910(45.5)	45.5	45.6	
≥8001	676(33.9)	35.5	32.2	
**Screen time** **(hrs./day)**				0.10
≤2	2038(84.9)	86.5	83.4	
2-3	239(10.0)	9.1	10.8	
>3	123(5.1)	4.5	5.8	
**Physical activity** ** (hrs./day)**				0.91
≤2	1942(80.9)	80.9	80.9	
2-3	292(12.2)	12.4	12.0	
>3	166(6.9)	6.7	7.1	
**Modes of travel to/from school**				**0.01**
Inactive (others)	1160(49.2)	52.0	53.5	
Active (walking/cycling)	1196(50.8)	48.0	46.5	
**Sugar-sweetened beverage** **(cps/wk.)**				**<0.01**
≤2	1497(63.4)	66.3	60.6	
2-5	256(10.8)	10.3	11.3	
5-7	483(20.5)	19.6	21.3	
>7	125(5.3)	3.8	6.8	
**Snacks**				
Healthy Snacks				0.68
Hardly eat	26(1.1)	1.1	1.1	
1-2/wk.	142(6.1)	5.5	6.7	
3-4/wk.	364(15.6)	15.8	15.5	
≥5/wk.	1795(77.2)	77.6	76.7	
Energy dense, nutrient poor				**0.03**
Hardly eat	142(6.1)	5.8	6.4	
1-2/wk.	964(41.3)	41.6	41.1	
3-4/wk.	779(33.4)	35.5	31.3	
≥5/wk.	447(19.2)	17.1	21.3	
Others				**0.01**
Hardly eat	40(1.7)	1.5	2.0	
1-2/wk.	242(10.4)	10.5	10.3	
3-4/wk.	530(22.8)	25.6	20.0	
≥5/wk.	1512(65.1)	62.4	67.7	

*P is for testing gender differences; Bold numbers refer to P<0.05.

^**△**^Weight status was defined according to WHO Child Growth Standards, BMI-for-age (5–19 years).

### Factors associated with weight status

On bivariate analyses (Table 2), screen time was significantly associated with children’s weight status. Surprisingly, physical activity was significantly associated with obesity (P = 0.01) with an unexpected direction. Modes of travel to school and consumption of SSB and snack were not associated with overweight & obesity or obesity.

**Table 2:** Factors associated with and overweight & obesity or obesity on bivariate analyses*

	**Non-overweight**				**Overweight & obese**				**Obese**			
	**n(row%)**	**OR**	**95% CI**	**P**	**n(row%)**	**OR**	**95% CI**	**P**	**n(row%)**	**OR**	**95% CI**	**P**
**Screen time** ** (hrs./day)**												
≤2 (ref)	1470(74.2)			**0.01**	510(25.8)			**0.01**	206(10.4)			**<0.01**
2-3	160(69.0)	0.77	0.57-1.04		72(31.0)	1.30	0.97-1.74		28(12.1)	1.18	0.78-1.80	
>3	76(63.3)	0.60	0.41-0.88		44(36.7)	1.67	1.14-2.45		28(12.1)	2.62	1.68-4.10	
**Physical activity** **(hrs./day)**												
≤2(ref)	1396(73.8)			0.32	495(26.2)			0.32	197(10.4)			**0.04**
2-3	197(70.4)	0.84	0.64-1.11		83(29.6)	1.19	0.90-1.57		42(15.0)	1.52	1.06-2.17	
>3	113(70.2)	0.84	0.59-1.19		48(29.8)	1.20	0.84-1.71		23(14.3)	1.43	0.90-2.28	
**Modes of travel to/from school**												
Inactive (others) (ref)	836(74.0)			0.33	293(26.0)			0.33	117(10.4)			0.22
Active (walking)	838(72.2)	0.91	0.76-1.10		322(27.8)	1.18	0.91-1.53		139(12.0)	1.10	0.91-1.32	
**SSB** ** (cps/wk.)**												
≤2(ref)	1086(74.3)			0.26	375(25.7)			0.26	152(10.4)			0.25
2-5	179(71.9)	0.88	0.65-1.19		70(28.1)	1.13	0.84-1.53		28(11.2)	1.09	0.71-1.67	
5-7	341(73.8)	0.97	0.77-1.24		121(26.2)	1.03	0.81-1.30		51(11.0)	1.07	0.76-1.50	
>7	81(66.4)	0.68	0.46-1.01		41(33.6)	1.47	0.99-2.17		20(16.4)	1.69	1.02-2.81	
**Snacks**												
Healthy Snacks												
Hardly eat (ref)	19(79.2)			**0.04**	5(20.8)			**0.02**	1(4.2)			0.11
1-2/wk.	87(63.0)	0.45	0.16-1.28		51(37.0)	2.38	0.78-7.21		24(17.4)	6.69	0.79-56.53	
3-4/wk.	258(73.1)	0.72	0.26-1.97		95(26.9)	1.46	0.50-4.29		40(11.3)	4.35	0.53-36.04	
≥5/wk.	1294(74.2)	0.76	0.28-2.03		451(25.8)	1.30	0.45-3.76		184(10.5)	3.95	0.49-32.21	
Energy dense, nutrient poor												
Hardly eat (ref)	94(69.6)			0.38	41(30.4)			0.37	19(14.1)			0.47
1-2/wk.	691(73.7)	1.22	0.82-1.81		247(26.3)	0.80	0.54-1.19		103(11.0)	0.74	0.44-1.27	
3-4/wk.	568(75.0)	1.31	0.88-1.96		189(25.0)	0.74	0.49-1.11		76(10.0)	0.67	0.39-1.16	
≥5/wk.	310(71.3)	1.08	0.71-1.65		125(28.7)	0.88	0.58-1.36		52(12.0)	0.82	0.46-1.46	
Others												
Hardly eat (ref)	27(73.0)			0.95	10(27.0)			0.63	6(16.2)			0.66
1-2/wk.	176(74.3)	1.07	0.49-2.34		61(25.7)	0.81	0.35-1.88		29(12.2)	0.55	0.20-1.51	
3-4/wk.	378(74.0)	1.05	0.50-2.23		133(26.0)	0.96	0.43-2.16		53(10.4)	0.51	0.19-1.36	
≥5/wk.	1073(72.9)	0.10	0.48-2.08		399(27.1)	1.02	0.46-2.26		162(11.0)	0.54	0.21-1.41	

*Unadjusted odds ratios were calculated on bivariate analyses with Spearman Correlations.

Bold numbers refer to P<0.05.

We further investigated the relationship between screen time and consumption of SSB and snacks. Interestingly, screen time (hrs./day) was negatively associated with “Healthy Snacks” (R = −0.13, P < 0.01), for boys (R = −0.12, P < 0.01) and girls (R = −0.13, P < 0.01) respectively; but positively associated with “Energy dense, nutrient poor” snacks (R = 0.14, P < 0.01), for boys (R = 0.14, P < 0.01) and girls (R = 0.13, P < 0.01) respectively. A positive association was also found between SSB consumption (cps/wk.) and screen time (R = 0.19, P < 0.01), for boys (R = 0.18, P < 0.01) and girls (R = 0.20, P < 0.01) respectively.

After adjusting for age, gender, household income, modes of travel to/from school, screen time, physical activity, SSB and “Healthy” snack (P < 0.25) were entered into the models on multilevel analyses, for obesity or overweight & obesity (Table 3). Screen time was a significant factor associated with obesity. Compared with those spent >3 hrs./day on screen time, children who spent ≤2 hrs./day or between 2-3 hrs./day were significantly less likely to be obese (AOR = 0.34, 95% CI: 0.20-0.60, P < 0.01; AOR = 0.41, 95% CI: 0.20-0.84, P = 0.02 respectively). There were other significant factors associated with obesity. These factors were examined for overweight & obesity. In addition, children spent ≤2 hrs./day on screen time were less likely to become overweight or obesity, compared with >3 hrs./day (AOR = 0.62, 95% CI: 0.38-0.99, P < 0.05).

**Table 3:** **Association between screen time and obesity or overweight & obesity using multilevel models**

		**Model A**		**Model B**		**Model C**		**Model D**		**Model E**	
		**AOR (95% CI)**	**P**	**AOR (95% CI)**	**P**	**AOR (95% CI)**	**P**	**AOR (95% CI)**	**P**	**AOR (95% CI)**	**P**
**Obesity**	**Screen time** **(hrs./d)**										
	≤2	0.34(0.20-0.60)	**<0.01**	0.35(0.20-0.62)	**<0.01**	0.34(0.20-0.60)	**<0.01**	0.36(0.21-0.63)	**<0.01**	0.37(0.21-0.66)	**<0.01**
	2-3	0.41(0.20-0.84)	**0.02**	0.39(0.19-0.81)	**0.01**	0.40(0.19-0.82)	**0.01**	0.40(0.20-0.83)	**0.02**	0.38(0.18-0.79)	**0.01**
	>3 (ref)										
**Overweight & Obesity**	**Screen time** **(hrs./d)**										
	≤2	0.62(0.38-0.99)	**<0.05**	0.62(0.38-1.01)	0.05	0.62(0.38-0.99)	**<0.05**	0.63(0.39-1.02)	0.06	0.63(0.39-1.03)	**0.07**
	2-3	0.81(0.45-1.44)	**0.45**	0.80(0.45-1.42)	**0.43**	0.81(0.46-1.45)	**0.46**	0.81(0.45-1.44)	**0.45**	0.81(0.45-1.44)	**0.45**
	>3 (ref)										

Model A. adjusted for age, gender, household income, modes of travel to/from school;

Model B. adjusted for those included in Model A + physical activity;

Model C. adjusted for those included in Model A + snack and/or screen time;

Model D. adjusted for those included in Model A + SSB;

Model E. adjusted for those included in Model A + snack + SSB + physical activity.

Bold numbers refer to P<0.05.

## Discussion

This study was one of few studies conducted in China in exploring the associations of lifestyle factors with obesity in children. The prevalence of obesity was 11.2% (14.4% for boys and 8.0% for girls), which was consistent with other research in China [15]. The combined prevalence for overweight and obesity was 26.8% overall, 36.2% in boys and 17.4% in girls. Screen time was a significant risk factor for overweight and obesity. However, physical activity and consumptions of SSB and snacks were not significantly associated with overweight or obesity.

### Relationship between physical activity, screen time and weight status

Some previous research found that total volume of physical activities was significantly lower in overweight and obese children, while levels of sedentary behaviors were significantly higher among them [16]. A study suggested that in both lean and obese youths, physical activity and sedentary behavior mainly affect energy balance through their impact on energy intake [17]. Wadden [18] found that physical activity alone is of limited benefit in inducing weight loss, thus obese individuals should be encouraged to exercise, at least in the short term, for the sake of improving their cardiovascular health, rather than for inducing weight loss.

Studies also found that increased weight status was more strongly and consistently associated with screen time than physical activity [19], which is consistent with our result. It is suggested that screen time may lead to increased energy intake; physical activities do not always offset the impact of screen time on the increase of body mass. Television viewing has been found to be associated with snack behaviors, high exposure to advertisements for energy dense foods [20]. Television viewing habits and acquired food preferences are potentially entrenched by adolescence, which will impact children’s health in later life. Our findings suggest that interventions targeting screen time reduction may be more effective for reducing overweight and obesity among children.

### Relationship between consumptions of SSB and snacks and weight status

A study in China suggested that regular SSB consumption was positively related to obesity and abdominal obesity, children who regularly consumed SSB were more prone to becoming obese than those who regularly drank milk [21]. Previous studies from other countries have also shown significant, positive associations between SSB consumption and weight gain among children [22,23]. However, a systematic review by Gibson [24] concluded that SSB is by nature a source of energy, but there is little evidence they are more obesogenic than any other source of energy. In our study, SSB consumption was significantly associated with screen time, and children consuming ≤2 cps/wk. SSB were less likely to be obese compared with those consuming >7cps/wk. (AOR = 0.52, 95% CI: 0.28-0.97, P = 0.04).SSB was inversely associated with obesity, which is likely due to the cross-sectional study design, i,e, those overweight children might have reduced SSB consumption. SSB might be an effective modifier in the relationship between screen time and obesity, and further research is needed to confirm.

Other similar research suggested that consumptions of foods and beverages during TV viewing might partially mediate the association between TV time and abdominal and central obesity [25,26]. The snack consumption was not found to have significant associations with obesity, although negative association existed between screen time and “Healthy Snacks” (R = −0.13, P < 0.01); positive association was found between screen time and “Energy dense, nutrient poor” snacks (R = 0.14, P < 0.01). Thorp et al. [27] recently found that adults in the high TV time/high snack food category were more likely to be obese, compared to those in the low TV time/low snack food category. It is plausible that the reason why we did not find significant results was that we could not differentiate from the FFQ whether children self-reported snack intake was consumed during TV viewing. Besides, children who were overweight or obese tend to under-report their snack intake [27], which might result in an underestimation of the strength of association between snack intake and obesity. Overall, the inconsistencies found in different studies suggest that more research, particular intervention studies are required.

One of the main strengths of this study was the use of a relative large sample size of primary school aged children. We used validated measurement tools for assessing the study factors. The strengths also included the use of multilevel models in examining the factors associated with obesity taking into account several confounders.

Limitations of the study include the cross-sectional study design, thus, causal relationships cannot be concluded. The generalizability of the study findings could be limited due to the survey sample coming from the Hongkou District, Shanghai only. The study findings were limited by child–parent self-reported measures including weight and height, physical activity, screen time and dietary habits. The study findings could be limited by excluding the dietary intake in the analyses as possibly dietary behaviors may differ in children with higher and lower screen time. Further, we were not able to tease out, using the FFQ, whether snack intake was consumed during their screen time.

## Conclusions

In conclusion, prevalence of childhood obesity in major cities like Shanghai in China has been increasing rapidly over the past decade. About a quarter of school-aged children are overweight or obese in Shanghai. Screen time is associated with childhood obesity, needs to be focused for obesity prevention in school age children in China.

## Abbreviations

SSB: Sugar-sweetened beverages
BMI: Body mass index
FFQ: Food frequency questions
AORs: Adjusted odds ratios
CI: Confidence intervals

## References

[1] WHO Regional Office for Europe (2012). Do surgical interventions to treat obesity in children and adolescents have long- versus short-term advantages and are they cost-effective?. HEN synthesis report.

[2] Burkhalter TM, Hillman CH (2011). A narrative review of physical activity, nutrition, and obesity to cognition and scholastic performance across the human lifespan. Adv Nutr.

[3] De Onis M, Blossner M, Borghi E (2010). Global prevalence and trends of overweight and obesity among preschool children. Am J Clin Nutr.

[4] Tang J, Chen SK, Luo JS, Fan X, Feng Y (2011). Epidemiologic status and influencing factors of obesity in children and adolescents. CJCHC.

[5] Ji CY, Chen TJ (2013). Empirical changes in the prevalence of overweight and obesity among Chinese students from 1985 to 2010 and corresponding preventive strategies. Biomed Environ Sci.

[6] Gubbels JS, van Assema P, Kremers SP (2013). Physical Activity, Sedentary Behavior, and Dietary Patterns among Children. Curr Nutr Rep.

[7] Ottevaere C, Huybrechts I, Benser J, De Bourdeaudhuij I, Cuenca-Garcia M, Dallongeville J (2011). Clustering patterns of physical activity, sedentary and dietary behavior among European adolescents: The HELENA study. BMC Public Health.

[8] Oza-Frank R, Zavodny M, Cunningham SA (2012). Beverage displacement between elementary and middle school, 2004–2007. J Acad Nutr Diet.

[9] Strong WB, Malina RM, Blimkie CJR, Daniels SR, Dishman RK, Gutin B (2005). Evidence Based Physical Activity for School-age Youth. J Pediatr.

[10] Song Y, Zhang X, Ma D, Zhang B, Hu PJ, Dong B (2012). Behavioral risk factors for overweight and obesity among Chinese primary and middle school students in 2010. Chin J Prev Med.

[11] Zhang J, Seo DC, Kolbe L, Middlestadt S, Zhao W (2012). Associated trends in sedentary behavior and BMI among Chinese school children and adolescents in seven diverse Chinese provinces. Int J Behav Med.

[12] Lu X, Shi P, Luo CY, Zhou YF, Yu HT, Guo CY (2013). Prevalence of hypertension in overweight and obese children from a large school-based population in Shanghai, China. BMC Public Health.

[13] Hardy LL, King L, Espinel P, Okely AD, Bauman A (2011). Methods of the NSW Schools Physical Activity and Nutrition Survey 2010 (SPANS 2010). J Sci Med Sport.

[14] WHO Child Growth Standards (2008). Training Course on Child Growth Assessment.

[15] Li H (2009). The growth status of Chinese children:trends in nutrition and development. Chin J Evid Base Pediatr.

[16] Tremblay MS, LeBlanc AG, Kho ME, Saunders TJ, Larouche R, Colley RC (2011). Systematic review of sedentary behaviour and health indicators in school-aged children and youth. Int J Behav Nutr Phys Act.

[17] Thivel D, Saunders TJ, Chaput JP (2013). Physical activity in children and youth may have greater impact on energy intake than energy expenditure. J Nutr Educ Behav.

[18] Wadden TA, Webb VL, Moran CH, Bailer BA (2012). Lifestyle modification for obesity: new developments in diet, physical activity, and behavior therapy. Circulation.

[19] Maher C, Olds TS, Eisenmann JC, Dollman J (2012). Screen time is more strongly associated than physical activity with overweight and obesity in 9- to 16-year-old Australians. Acta Paediatr.

[20] Boyland EJ, Halford JC (2013). Television advertising and branding. Effects on eating behaviour and food preferences in children. Appetite.

[21] Shang XW, Liu AL, Zhang Q, Hu XQ, Du SM, Ma J (2012). Report on childhood obesity in China (9): sugar-sweetened beverages consumption and obesity. Biomed Environ Sci.

[22] Grimes CA, Riddell LJ, Campbell KJ, Nowson CA (2013). Dietary salt intake, sugar-sweetened beverage consumption, and obesity risk. Pediatrics.

[23] American Academy of Pediatrics (2004). Soft drinks in schools. Pediatrics.

[24] Gibson S, Neate D (2007). Sugar intake, soft drink consumption and body weight among British children: further analysis of National Diet and Nutrition Survey data with adjustment for under-reporting and physical activity. Int J Food Sci Nutr.

[25] Cleland VJ, Schmidt MD, Dwyer T, Venn AJ (2008). Television viewing and abdominal obesity in young adults: is the association mediated by food and beverage consumption during viewing time or reduced leisure-time physical activity?. Am J Clin Nutr.

[26] Thomson M, Spence JC, Raine K, Laing L (2008). The association of television viewing with snacking behavior and body weight of young adults. Am J Health Promot.

[27] Thorp AA, McNaughton SA, Owen N, Dunstan DW (2013). Independent and joint associations of TV viewing time and snack food consumption with the metabolic syndrome and its components; a cross-sectional study in Australian adults. Int J Behav Nutr Phys Act.

